# Immunogenicity of Rabies Virus G-Protein mRNA Formulated with Muscle-Targeting Lipid Nanoparticles in Mice

**DOI:** 10.3390/vaccines13030217

**Published:** 2025-02-22

**Authors:** Qin Li, Huarong Bai, Xueliang Yu, Qiang Liu, Rongkuan Hu

**Affiliations:** Starna Therapeutics Co., Ltd., Suzhou 215123, Chinabaihuarong@starnatx.com (H.B.);

**Keywords:** rabies virus, mRNA vaccine, muscle delivery, lipid nanoparticles

## Abstract

Background: Rabies is a preventable zoonotic disease caused by the rabies virus (RABV) with a high mortality rate. Most vaccines on the market or under development have issues, such as low single-dose neutralization titer, complex processes, and high costs. During the COVID-19 pandemic, the successful development of mRNA vaccines opened up a new avenue for preventive vaccines. As a new technology, mRNA has higher scalability. Methods: In this study, we designed an mRNA encoding the RV-G protein, encapsulated by our own muscle-targeting lipid nanoparticles (LNPs), and evaluated the expression of the RV-G protein in vitro, its immunogenicity, and its protection against virus infection in vivo. Results: The results show that RV-G mRNA was significantly expressed in vitro. High Virus-IgG binding titers and virus-neutralizing antibody titers (VNT) were induced by immunization with RV-G mRNA-LNP. Additionally, our results showed that the RV-G mRNA vaccine is better than commercially available vaccines in mice. Conclusions: Our research highlights the potential of the mRNA-LNP platform in developing next-generation rabies vaccines.

## 1. Introduction

Rabies is a fatal disease caused by the rabies virus (RABV). RABV invades the host’s central nervous system and causes pathological changes. Once the virus takes effect and the symptoms appear, it cannot be eliminated by the immune system or any drugs, and the fatality rate is nearly 100%. Traditional rabies vaccines have been a cornerstone in the prevention of this deadly disease for decades.

The RABV has a single-strand RNA genome and five structural proteins, including glycoprotein (G), nucleocapsid protein (N), phosphoprotein (P), matrix protein (M), and viral RNA polymerase (L) [1]. Among them, the G protein is the only surface-exposed viral protein in RABV virions. It is responsible for binding to the host cell receptors and facilitating viral entry into the host cells. The G protein also elicits the immune response in the host and is the main target protein that stimulates virus-neutralizing antibodies [2]. All the other components play crucial roles in the virus’s replication and pathogenesis.

The disease has been an important public health problem with no effective treatment to date. Currently, prevention is mainly through vaccination. There are mainly two types of commercially available rabies vaccines: inactivated and attenuated rabies vaccines. Inactivated rabies vaccines are the most common and widely used around the world. They are produced by growing the rabies virus in cell cultures, such as human diploid cells (HDC), chicken embryo fibroblasts, and Vero cells [3,4,5,6]. After growth, the virus is inactivated using chemicals, and it is ensured that the virus is no longer capable of causing the disease. However, the cost of inactivated vaccines is high, and the dosage process is relatively cumbersome. The main reason is the low titer of the rabies virus produced, which requires multiple injections to reach a higher-level titer to obtain satisfactory protective efficacy. Besides, attenuated rabies vaccines are mainly used for wildlife and animal vaccination. To date, novel DNA vaccines, mRNA vaccines, and viral vector vaccines have been evaluated to protect against rabies infection in preclinical settings, but these vaccines have not been approved for use in humans yet [7,8,9].

Messenger RNA (mRNA) vaccines represent a revolutionary approach that has received significant attention both from academia and industry, especially during the COVID-19 pandemic, showing great promise and opening up new avenues for vaccine development and pandemic preparedness [10,11]. mRNA vaccines work by introducing mRNA molecules into the body, where they serve as a template to produce specific antigens. These antigens then trigger an immune response, enabling the body to recognize and defend against the virus in the future. Unlike traditional vaccines, mRNA vaccines do not contain live or inactivated viruses, reducing the risk of causing the disease and having the potential for rapid development and production, as the manufacturing process does not rely on cell cultures. Currently, there are several studies in rabies mRNA vaccine development. CureVac conducted a preclinical study on the protective efficacy of its mRNA rabies vaccine in monkey models. The results showed that compared with the inactivated vaccine, Rabipur, the mRNA vaccine could induce higher levels of neutralizing antibodies and produce more extensive reactive antibody protection [12]. LVRNA001, the mRNA rabies vaccine developed by Liverna, could provide 100% safe and effective immune protection with only two doses [13]. Another study introduced a newly designed LNP-mRNA rabies vaccine, which demonstrated excellent performance in rhesus monkeys and mice with low dosage [14]. The strong immune response induced by mRNA vaccines suggests that they have great potential for use in both human and animal rabies prevention and can potentially achieve better immunogenicity and protective effects with fewer doses.

In this study, we developed an mRNA-based rabies vaccine. This approach yielded high titers of virus-neutralizing antibodies and Virus-IgG binding antibodies in mice, and it demonstrated effective protective efficacy in subsequent challenge experiments.

## 2. Materials and Methods

### 2.1. Cell Lines

HEK293T cells, sourced from the Chinese Academy of Sciences Cell Bank in Beijing, China, and cos.7 cells, donated by Westlake University, were cultured in DMEM (HyClone, Logan, UT, USA), supplemented with 10% FBS (Gibco, Carlsbad, CA, USA), and 1% Penicillin–Streptomycin (Gibco, Carlsbad, CA, USA). Both of the cell lines were maintained under sterile conditions and incubated at a temperature of 37 °C with 5% CO_2_.

### 2.2. mRNA Design and Preparation

UTR pairs were selected from the proprietary UTR library of Starna. The coding sequence of the RV-G mRNA was obtained by performing simple codon optimization on the RV-G sequence retrieved from GenBank (CTN-1 strain, Accession Number: AY009100.1). The construction of the DNA template for in vitro transcription was carried out by GeneScript (Nanjing, China). For mRNA preparation, the process started with in vitro synthesis using the T7 High Yield RNA Transcription Kit (Thermo, Waltham, MA, USA, K0441). The resulting mRNA was then purified using the Monarch RNA Cleanup Kit (NEB, Ipswich, MA, USA).

### 2.3. Lipid Nanoparticle Preparation

RV-G mRNA-LNP formulations were crafted using a proprietary method. The lipid components, each with a defined molar ratio, were dissolved in ethanol, and the RNA was prepared in a 10 mM citrate buffer at pH 4.0. The synthesis process employed microfluidic technology, with an ethanol-to-water phase volume ratio set at 3:1 and a flow-rate ratio of 1:3. Subsequently, a dialysis step was performed in 1× PBS for 2 h to refine the formulation.

### 2.4. mRNA Transfection

HEK-293 cells were seeded in 96-well or 6-well plates one day before transfection. On the day of transfection, the cells were treated with 100 ng or 4 µg of luciferase or RV-G mRNA using Lipo2000 as the transfection reagent. After a 24 h incubation, the cells were harvested. Luciferase activity was measured by luciferase assay, and the expression of the RV-G protein was subsequently detected by Western blot and FACS analysis.

### 2.5. Luciferase Assay

The luciferase assay was carried out according to the Dual-Luciferase Reporter Assay Kit (Promega, Madison, WI, USA, E1910). Cells transfected with luciferase mRNA using Lipo2000 were harvested, the supernatant was removed, and the cells were rinsed once with 100 µL of PBS buffer, after which the buffer was discarded. Then, 105 µL of prediluted lysis buffer was applied to cover the cells and incubated at room temperature for 15 min. Subsequently, the 96-well plate was centrifuged at 13,000 rpm for 5 min. The supernatant was carefully transferred to a 96-well detection plate, with 30 µL aliquoted per well, and each sample was assayed in triplicate. Finally, 30 µL of luciferin substrate was added to each well in the dark, and the luminescence values were read using a microplate reader (TECAN SPARK, Mannedorf, Switzerland).

### 2.6. Western Blot

Cells transfected with RV-G mRNA using Lipo2000 were harvested, and the total protein concentration was measured using the Bradford assay. Subsequently, 1× SDS loading buffer was added to the samples, which were then subjected to SDS-PAGE and transferred onto PVDF membranes. The membranes were blocked with TBST containing 5% (*w*/*v*) non-fat dry milk and incubated with primary antibody (Absolute antibody, Ab02097-3.0) for 2 h at room temperature. After that, they were incubated with horseradish peroxidase (HRP)-conjugated secondary antibodies (Jackson ImmunoResearch, Lancaster, PA, USA, 115-035-003) for 1 h at 37 °C. After three washes with 1× PBST, the blots were developed using a developing solution (Tanon, Shanghai, China, 180-5001). The resulting images were captured using a Tanon 4200 imaging system.

### 2.7. Mice Immunization Procedure and Challenge

After a 3-day acclimation period, groups of six 6–8-week-old Balb/C mice were intramuscularly (i.m.) injected with 50 µL of different RV-G mRNA-LNP formulations, each containing 5 µg of mRNA. Plasma and serum samples were collected at 7 and 14 days post-injection for serological testing.

For the pre-exposure protection study of RV-G mRNA-LNP, mice were intracerebrally injected with 100-fold the 50% lethal dose (LD50) of RABV CVS-24 strain 14 days after the immunization procedure. Serum was collected on day 14 for neutralization assays and to determine Virus-IgG binding titers.

In the post-exposure protection study of RV-G mRNA-LNP, mice were intracerebrally injected with 100-fold of the 50% lethal dose (LD50) of RABV CVS-24 strain 6 h before the immunization procedure on day 0. Serum was collected on day 14 for neutralization assays. After the challenge infection, the survival rates and body weight changes in the mice were monitored daily.

### 2.8. Inflammation Cytokines and Biochemistry Test

Serum samples were analyzed for inflammation biomarkers (IFNγ, IL-6, and TNF-α) using the ELISA kit (Abcam, Cambridge, UK, #ab282874, #ab222503, #ab208348). Plasma biochemical tests were carried out using an automatic biochemistry analyzer (HITACHI 7100+ISE).

### 2.9. Enzyme-Linked Immunosorbent Assay (ELISA)

IgG titers in the immunized sera were assessed using an ELISA method. ELISA plates were coated with recombinant the G antigen of Rabies Virus (final concentration: 2 µg/mL) (ABMAX Biotech, Beijing, China, AK100H1) and incubated at 4 °C overnight. After being washed with PBST twice, the plates were incubated with diluted serum following a blocking step with PBS containing 2% BSA for 2 h at 37 °C. Subsequently, the plates were incubated for an additional 2 h at 37 °C with a rabbit antimouse secondary antibody conjugated to HRP (Abcam, Cambridge, UK, ab6728) to measure the specific binding of antibodies to the RV-G protein using a TMB substrate.

### 2.10. Neutralization Assay

Rabies virus challenge virus standard strain (CVS-11) was propagated on BSR cells and utilized in the experiments, following the Reed–Muench method. The standard serum (obtained from the National Institute for Biological Standards and Control, Hertfordshire, UK) was diluted to a concentration of 0.5 IU/mL with DMEM. A volume of 100 μL DMEM medium was added to each well of a 96-well plate, followed by the addition of 50 μL of the tested serum, standard serum, or negative control serum to the first column. Subsequently, a 3-fold gradient dilution was carried out across the plate. The test serum was diluted through column 9, while the standard and negative sera were diluted through column 6, with 50 μL being discarded from the final column. The CVS-11 virus was diluted to a concentration of 100 FFU per 50 μL with DMEM, and 50 μL of this virus dilution was added to each well containing the sera. Then, 100 μL of a BSR cell suspension containing 2 × 10^4^ cells were added to each well and incubated in a 5% CO_2_ incubator at 37 °C for 60 h.

The culture medium was then removed from the 96-well plate, and the wells were fixed with precooled acetone at −20 °C for 30 min and washed three times with PBS. The plate was incubated with a FITC-conjugated antibody specific for the rabies virus nucleoprotein (Novus, Centennial, CO, USA, NBP3-33166F) at 37 °C for 45 min. After discarding the antibody incubation solution and washing three times with PBS, the cells were observed under a fluorescence microscope. The absence of fluorescence was recorded as “−”, while the presence of one or more fluorescent cells was recorded as “+”. The neutralizing antibody titers were determined accordingly. The virus-neutralizing antibody titers (VNT) were calculated using the Reed–Muench method.

### 2.11. Data Analysis

Data analysis was conducted using GraphPad Prism version 8.0.2 for Windows (GraphPad Software, Inc., La Jolla, CA, USA). T-tests were employed to assess statistical significance. Kaplan–Meier survival curves were utilized to evaluate the statistical significance of survival rates post challenge. The thresholds for statistical significance are denoted as follows: * for *p* < 0.05, ** for *p* < 0.01, *** for *p* < 0.001, and **** for *p* < 0.0001; “ns” indicates no significant difference.

## 3. Results

### 3.1. Optimization of RV-G mRNA

**Figure 1:**
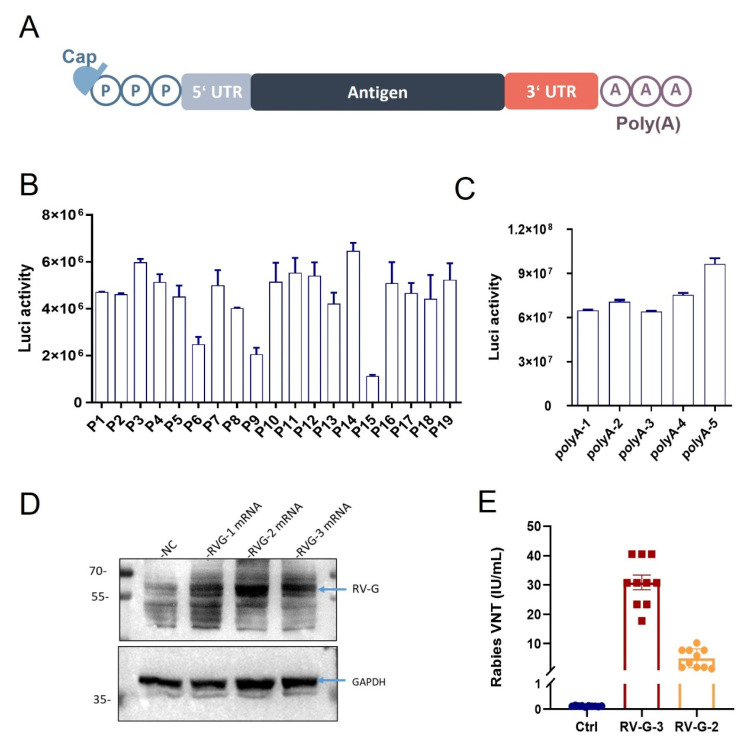
Optimization of RV-G mRNA sequence: (**A**) key elements of synthetic mRNA; (**B**) screening results of UTR pairs based on the luciferase sequence; (**C**) results of PolyA optimization based on the luciferase sequence; (**D**) expression of RV-G protein for the three RV-G RNA variants; (**E**) virus-neutralizing antibody titers in the serum.

Based on the key elements of mRNA as shown in Figure 1A, we optimized the RV-G mRNA sequence. We initially screened for appropriate UTR pairs in the proprietary UTR library of Starna. These UTR pairs encompassed natural sequences, optimized sequences, AI-generated sequences, and de novo-designed sequences. The complete list of UTR pairs can be found in Table S1. After selecting the UTR pairs, we employed the luciferase sequence as a coding region for initial screening, capitalizing on its straightforward manipulation. Different UTR pairs were incorporated into luciferase mRNA, which was then transfected into cos.7 cells, and the luciferase (luc) activity was measured. The outcomes revealed excellent luciferase expression across all UTR pairs (Figure 1B), indicating that all UTR pairs were highly effective when integrated into luciferase mRNA, with P14 being the best pair.

Subsequently, we screened different types of polyA for their application. The candidates included full-length polyA and fragmented polyA. The specific sequences are shown in Table S1. Results show that the fragmented polyA showed a higher expression level (Figure 1C). So far, we screened out the preferred UTR pair and the polyA.

Afterward, we replaced the luciferase sequences with three optimized RV-G RNA sequences (Table S2). These RV-G RNA variants were transfected into HEK293T cells, and Western blot analysis was employed to detect RV-G expression. The findings indicated that the expression levels of RV-G in RV-G-1, RV-G-2, and RV-G-3 were markedly higher than those of the blank control in HEK-293T cells, confirming the successful expression of the RV-G protein by these RV-G RNA constructs (Figure 1D). And in the in vivo experiments, the neutralizing antibody titers induced by RV-G-3 RNA were much higher than those provoked by RV-G-2 RNA after a single injection of 5 µg (Figure 1E). Therefore, we selected RV-G-3 RNA, which demonstrated better protein expression and higher neutralizing antibody titers, for further research.

### 3.2. Muscle-Targeting LNP Formulation Screening

**Figure 2:**
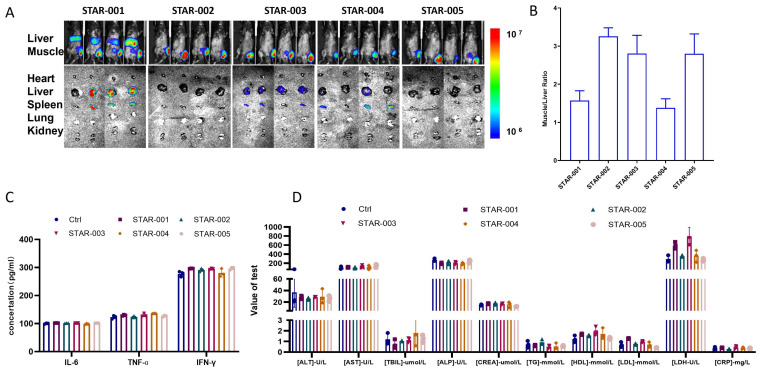
Muscle-targeting LNP formulation screening: (**A**) Mice were injected with 1 µg of LNP-mRNA encoding luciferase; 6 h after injection, luciferase expression was visualized in vivo. Then, the mice were sacrificed, and the heart, liver, spleen, lung, and kidney were photographed under fluorescence. (**B**) Muscle to liver ratio was calculated. (**C**) Results of inflammatory cytokines assay. (**D**) Biochemical test results of the plasma.

To screen muscle-targeting LNP formulations, we introduced five distinct ones and evaluated the in vivo expression and safety-related immune responses. STAR-001, a traditional liver-targeting lipid nanoparticle (LNP), was used as a positive control, while for the other LNPs, cationic lipid was used as previously described [15,16]. Mice were intramuscularly injected with mRNA encoding luciferase (Luc) encapsulated within these varying LNP formulations. Six hours post-injection, luciferase expression in the mice was monitored by a bioluminescence imaging system. The findings revealed that all mice injected with Luc mRNA-LNP demonstrated luciferase expression, with the STAR-001 formulation showing the highest efficacy in the liver (Figure 2A). In this research, we focused on developing mRNA vaccine formulations optimized for intramuscular injection rather than liver targeting. We hoped to observe more enrichment at the site of muscle injection. As shown in Figure 2B and Table 1, apart from the differences in the zeta potential, the particle sizes and polydispersity indices (PDIs) of these four LNP components did not vary significantly. However, the STAR-002 formulation had the highest muscle-to-liver ratio.

To further assess the tolerability and safety of the LNP formulations, blood samples were collected one week after injection and analyzed for safety-related indicators. The data indicated that the serum concentrations of inflammatory cytokines (IL-6, TNF-α, and IFN-γ) and the plasma’s biochemical test results for most mice were similar to those in the mice injected with PBS (Figure 2C,D). Moreover, no significant weight loss was observed throughout this study. Collectively, these results suggest that all the LNP formulations mediate effective mRNA delivery and expression and exhibit excellent tolerability and safety profiles. The STAR-002 formulation, exhibiting superior muscle-targeting efficiency, was chosen for further development.

### 3.3. RV-G mRNA-LNP Induces Strong Humoral Responses in Mice

**Figure 3:**
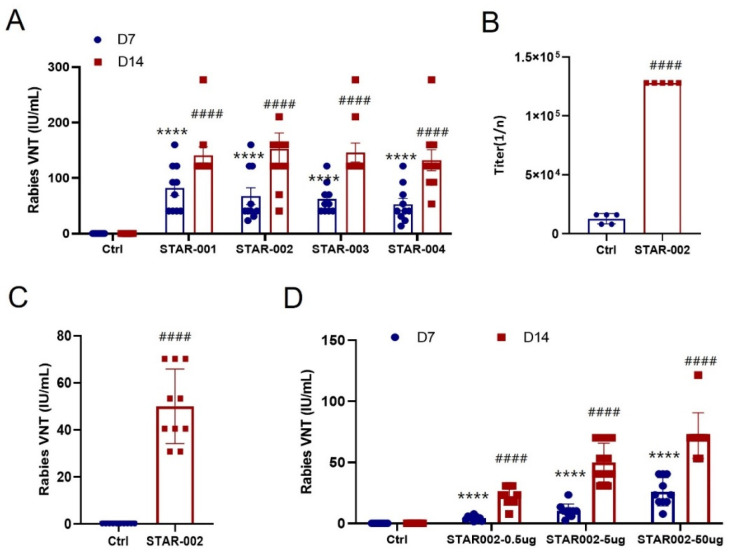
Antigen-specific antibody response in mice: (**A**) virus-neutralizing antibody titers in the serum of vaccinated mice (50 µg) detected by ELISA in the four superior formulations; (**B**) analysis of the antigen-specific antibody response in mice administered a 5 µg dose in serum collected on day 14; (**C**) virus-neutralizing antibody titers in the serum of the 5 µg dose in serum collected on day 14; (**D**) virus-neutralizing antibody titers in the serum of mice with three doses, collected on day 7 and day 14 (****, *p* < 0.001 compared with the Ctrl group on day 7; ####, *p* < 0.001 compared with the Ctrl group on day 14).

We have demonstrated that the optimized LNP formulations effectively mediate mRNA delivery and expression, along with good tolerability and safety. Additionally, through rigorous UTR pairs screening, we have identified the confirmed RV-G mRNA sequence. Here, we encapsulated the confirmed RV-G mRNA in the four superior formulations to obtain the optimal RV-G mRNA-LNP. BALB/c mice were immunized with these RV-G mRNA-LNPs on day 0, and blood samples were collected via the orbital venous plexus on days 7 and 14 for serological studies. Neutralization assay was performed, revealing that the four LNP formulations induced significantly higher virus-neutralizing antibody titers (VNT) on days 7 and 14 post-injection compared with the PBS group (Figure 3A). Notably, the STAR-002 formulation stood out with higher VNTs and less variability among groups. Consequently, we selected this formulation for further investigations.

Subsequently, we evaluated the in vivo immune response of the selected RV-G mRNA-LNP. BALB/c mice were immunized with 0.5, 5, and 50 µg doses of RV-G mRNA-LNP on day 0. Serological studies were conducted using blood samples collected via the orbital venous plexus on days 7 and 14 post-injection. ELISA was used to measure Virus-IgG binding titers, and neutralization assay was performed. Results show that on day 14, mice in the 5 µg RV-G mRNA-LNP group exhibited higher virus-neutralizing antibody titers (VNT) and Virus-IgG binding titers (1:128,000) than the control group (Figure 3B,C). The VNT titers on day 7 ranged from 4.5 IU/mL in the 0.5 µg dose group to 26 IU/mL in the 50 µg dose group. By day 14, these titers increased to range from 22.4 IU/mL in the 0.5 µg dose group to 71.9 IU/mL in the 50 µg dose group, demonstrating a positive dose-response relationship (Figure 3D). These findings suggest that mRNA-LNP encapsulated with RV-G mRNA can elicit higher levels of Virus-IgG binding titers and virus-neutralizing antibody titers (VNT) against the RV-G protein.

### 3.4. The Pre-Exposure Protection of RV-G mRNA-LNP and Its Capability of Competing with Licensed Vaccines in Mice

To assess the prophylactic capacity of RV-G mRNA-LNP in vivo, we conducted an experiment using 6–8-week-old Balb/C mice (*n* = 10, with an equal number of males and females). The mice were intracerebrally infected with 100-fold LD50 CVS-24 strain 14 days after receiving a single 5 µg dose of immunization. A licensed vaccine served as a positive control.

**Figure 4:**
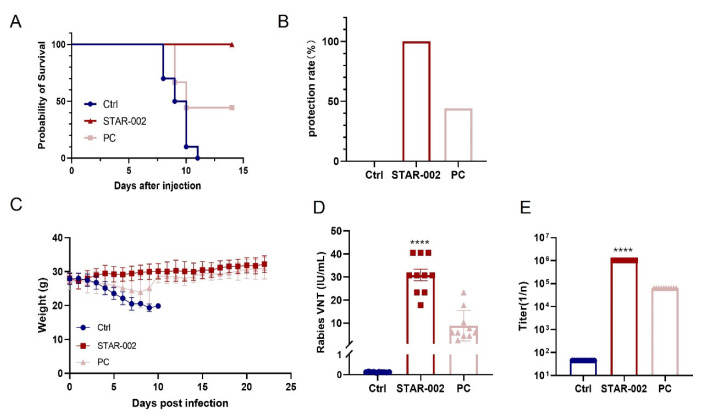
Pre-exposure protection of RV-G mRNA vaccine in mice: (**A**) Kaplan–Meier survival curves; (**B**) protection rate statistical chart; (**C**) body weight changes in mice; (**D**) virus-neutralizing antibody titers in the serum; (**E**) Virus-IgG binding titers in serum (****, *p* < 0.001 compared with PC group).

The results are striking: all mice in the RV-G mRNA-LNP group survived, whereas 6 out of 10 mice in the positive control group succumbed. This indicates a 100% protection rate for the RV-G mRNA-LNP group, compared with a 40% protection rate for the positive control group (Figure 4A,B). Additionally, we observed varying body weight changes among the groups. The RV-G mRNA-LNP group exhibited the least weight fluctuation, followed by the positive control group, which experienced a 10-day period of weight loss post-infection before gradually recovering. In contrast, the control groups continued to lose weight until they died (Figure 4C).

We also compared the immunogenicity of RV-G mRNA-LNP with that of the licensed vaccine. A single 5 µg dose of RV-G mRNA-LNP induced virus-neutralizing antibody titers (VNT) of 31 IU/mL, significantly higher than the 9 IU/mL induced by the licensed vaccine. Similarly, for Virus-IgG binding titers, the RV-G mRNA-LNP group showed a titer of 1:1,024,000, in contrast to the 1:64,000 of the licensed vaccine (Figure 4D,E).

These findings suggest that a single 5 µg dose of RV-G mRNA-LNP results in virus-neutralizing antibody titers (VNT) nearly six times the WHO standard of 0.5 IU/mL by day 14. This level is sufficient to induce protective antibody titers.

### 3.5. The Post-Exposure Protection of RV-G mRNA-LNP

The aforementioned results clearly demonstrate that RV-G mRNA-LNP could protect mice from CVS-24 infection following pre-exposure immunization. Building on these findings, we sought to determine if RV-G mRNA-LNP also offers protection post-exposure. For this, we used 6–8-week-old Balb/C mice (*n* = 10, with an equal number of males and females) and intramuscularly infected them with 50-fold LD50 CVS-24 6 h prior to administering a single 5 µg dose of the immunization. Formulation SM-102 served as a positive control.

**Figure 5:**
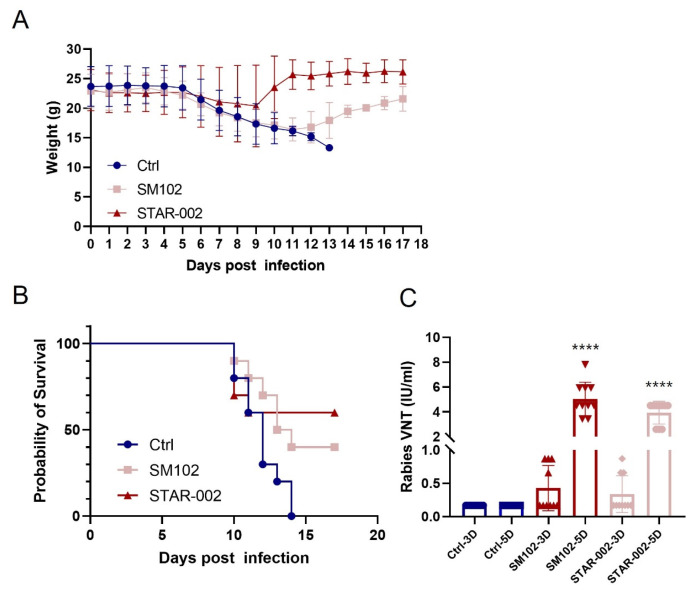
Post-exposure protection of RV-G mRNA vaccine in mice: (**A**) body weight changes in mice; (**B**) Kaplan–Meier survival curves; (**C**) virus-neutralizing antibody titers in the serum (****, *p* < 0.001 compared with SM102 group).

After infection, all groups experienced weight loss within the first 5 days; however, mice immunized with RV-G mRNA began to regain weight gradually, with the STAR-002 group demonstrating a more rapid recovery (Figure 5A). The survival rates among the groups varied significantly. The STAR-002 group lost four mice, resulting in a 60% survival rate, while the SM-102 group lost six mice, yielding a 40% survival rate. In contrast, all mice in the control group succumbed to rabies within 14 days of infection (Figure 5B). Additionally, a single dose of STAR-002 and SM-102, both encapsulating RV-G mRNA, induced high levels of virus-neutralizing antibody titers (VNT) 5 days post administration, which were significantly higher than the WHO standard of 0.5 IU/mL (Figure 5C). These outcomes highlight that the STAR-002 formulation outperformed SM-102, providing superior protection against viral infection in vivo.

## 4. Discussion

mRNA represents a highly promising multifunctional vaccination approach and revolutionizes the vaccine landscape, as underscored by its pivotal role in combating the COVID-19 pandemic. Two mRNA-based SARS-CoV-2 vaccines, mRNA-1273 (Moderna) and BNT162b2 (Pfizer-BioNTech), were swiftly developed, showcasing high safety and efficacy with relatively low costs [17,18]. This success has spurred research into applying mRNA technology to other infectious diseases, including respiratory syncytial virus (RSV), varicella zoster virus (VZV), and rabies virus.

The inactivated rabies vaccine is prevalent globally, playing a crucial role in preventing and controlling the spread of rabies. However, most inactivated rabies vaccines necessitate multiple doses to achieve high neutralizing antibody titers, which incur substantial costs and also pose challenges in terms of patient compliance. Thus, the development of innovative vaccines like mRNA vaccine is desirable to help reduce the burden of rabies.

In this study, we engineered an RV-G mRNA vaccine focused on the rabies virus glycoprotein, the sole certified neutralizing antibody binding epitope. In the beginning, by optimizing the UTRs of RV-G mRNA, we enhanced the mRNA’s stability and efficiency since UTRs are crucial for both the expression and half-life period (Figure 1B). Then, we optimized the polyA tails of different lengths and inserted sequences in the middle of the tail. The polyA tail is also crucial for the stability and translation efficiency of mRNA. Our new polyA tail effectively evades traditional patents and demonstrates similar stability and expression efficiency (Figure 1C). After that, we introduced a new muscle-targeted LNP formulation with our proprietary new cationic lipid, which avoids the leakage problem of traditional liver-targeted LNPs (Figure 2A,B). The safety profiles of these different LNP formulations are quite comparable (Figure 2C,D). Moreover, compared with the traditional liver-targeted lipid SM-102 (STAR-001), our new cationic lipid (STAR-002) can generate a stronger humoral immune response, especially in terms of neutralizing antibody level (Figure 3A). In Figure 3B–D, the RV-G mRNA, when encapsulated with STAR-002 LNP, induced high virus-neutralizing antibody titers (VNT) and IgG titers in a dose-dependent manner. Notably, the STAR-002 formulation provided 100% protection in pre-exposure challenge assays in mice, markedly outperforming the licensed vaccine’s 40% protection (Figure 4). In post-exposure challenge assays, a single dose induced 60% protection, surpassing the classic formulation SM102, which offered 40% protection. Moreover, a single 5 µg dose of this vaccine yielded neutralizing antibody titers nearly 200 times the WHO standard of 0.5 IU/mL (Figure 5). Compared with previously reported mRNA-LNP rabies vaccines developed by CureVac and Liverna, the vaccines in our study have two unique features. Apart from differences in the mRNA backbones, the muscle-targeted LNPs used here can generate a stronger humoral immunity compared with traditional liver-targeted LNPs. The latter may have off-target toxic side effects in the liver.

LNP-formulated mRNA can serve as a versatile platform technology [19], which can be manufactured efficiently through a standardized process. The simplicity and scalability of the LNP-formulated mRNA method, along with the ease of batch control, significantly reduce vaccine production costs. In conclusion, the encapsulation of RV-G mRNA as an antigen in LNP-based nanoparticles not only elicits virus-neutralizing antibody titers (VNT) but also achieves significant immune effects with a single injection. These attributes render the mRNA vaccine a potentially safe and cost-effective candidate for rabies vaccination.

## 5. Conclusions

This study first constructed a novel backbone for an mRNA vaccine by optimizing the UTRs and polyA tail. This framework is suitable for the development of various preventive mRNA vaccines. Subsequently, through a small screening process, we obtained a muscle-targeted LNP formulation. Compared with traditional liver-targeted LNPs, this new delivery system exhibits more potency in generating higher levels of neutralizing and IgG antibodies. Finally, we combined this backbone with RV-G antigen sequence encapsulated in a muscle-targeted LNP system to develop a rabies virus mRNA vaccine. This candidate demonstrates strong protective efficacy in mice and has promising prospects for future development.

## Figures and Tables

**Table 1:** Size, PDI, and zeta of LNP formulations.

LNP	Size (nm)	PDI	Zeta (mV)
STAR-001	104.20	0.054	−2.60
STAR-002	103.10	0.062	6.19
STAR-003	100.60	0.070	3.90
STAR-004	107.35	0.073	−7.45
STAR-005	101.40	0.148	1.43

## Data Availability

The data presented in this study are available upon request from the corresponding author.

## Supplementary Materials

The following supporting information can be downloaded at https://www.mdpi.com/article/10.3390/vaccines13030217/s1, Figure S1: Original images for WB; Table S1: UTR sequences for UTR pair screening; Table S2: sequences for the RV-G RNA.
