# Supplementary material for: Immunogenicity of Rabies Virus G-Protein mRNA Formulated with Muscle-Targeting Lipid Nanoparticles in Mice
Source: Vaccines (Basel). 2025 Feb 22;13(3):217. doi: 10.3390/vaccines13030217 (PMC11945611; doi:10.3390/vaccines13030217)
Supplement: Supplementary file 1 [file vaccines-13-00217-s001.zip › vaccines-3440088-supplementary.pdf]

Immunogenicity of Rabies Virus G-Protein mRNA Formulated with Muscle Targeting Lipid Nanoparticles in Mice

Supplemental data

Table S1. UTR sequences for UTR pair screening.

NO.	5UTR SEQ.	3UTR SEQ.
P1	AGGACCAACACAAACACACAAACACAACAAACACACAAACACACAAACACAACACAACACA	GCTCGCTTTCTTGCTGTCCAATTTCTATTAAAGGTTCCTTTGTTCCCTAAGTCCAACTACTAAACTGGGGGATATTATGAAGGGCCTTGAGCATCTGGATTCTGCCTAATAAAAAACATTTATTTTCATTGCAATTGCCATGTGTATGTGGGTTCGCCCACATACTCTGATGATCCCCAATCGTGGCGTGTCGGCCTGCTTCGGCAGGCACTGGCGCCGGGATCATTCATGGCAA
P2	AGGACCATCACATACATACATACACAACTAACTCACAATCACACTAACACTACACATCACA	GCTCGCTTTCTTGCTGTCCAATTTCTATTAAAGGTTCCTTTGTTCCCTAAGTCCAACTACTAAACTGGGGGATATTATGAAGGGCCTTGAGCATCTGGATTCTGCCTAATAAAAAACATTTATTTTCATTGCAATTGCCATGTGTATGTGGGTTCGCCCACATACTCTGATGATCCCCAATCGTGGCGTGTCGGCCTGCTTCGGCAGGCACTGGCGCCGGGATCATTCATGGCAA
P3	AGTTGTTAGTCTACGTGGACCGACAAAGACAGATTCTTTGAGGGAGCTAAGCTCAACGTAGTTCTAACAGTTTTTAATTAGAGAGCAGATCTCTG	GCTCGCTTTCTTGCTGTCCAATTTCTATTAAAGGTTCCTTTGTTCCCTAAGTCCAACTACTAAACTGGGGGATATTATGAAGGGCCTTGAGCATCTGGATTCTGCCTAATAAAAAACATTTATTTTCATTGCAATTGCCATGTGTATGTGGGTTCGCCCACATACTCTGATGATCCCCAATCGTGGCGTGTCGGCCTGCTTCGGCAGGCACTGGCGCCGGGATCATTCATGGCAA
P4	AGTTGAAAGTCTACGTGGACCGACAAAGACAGATTCACAGAGGGAGCTAAGCTCAACGTAGTTCTAACAGTACAACAACAGAGAGCAGATCTCTG	GCTCGCTTTCTTGCTGTCCAATTTCTATTAAAGGTTCCTTTGTTCCCTAAGTCCAACTACTAAACTGGGGGATATTATGAAGGGCCTTGAGCATCTGGATTCTGCCTAATAAAAAACATTTATTTTCATTGCAATTGCCATGTGTATGTGGGTTCGCCCACATACTCTGATGATCCCCAATCGTGGCGTGTCGGCCTGCTTCGGCAGGCACTGGCGCCGGGATCATTCATGGCAA
P5	GGGAAATAACACACAGAACAACACTAACAACAAATATAACA	GCTCGCTTTCTTGCTGTCCAATTTCTATTAAAGGTTCCTTTGTTCCCTAAGTCCAACTACTAAACTGGGGGATATTATGAAGGGCCTTGAGCATCTGGATTCTGCCTAATAAAAAACATTTATTTTCATTGCAATTGCCATGTGTATGTGGGTTCGCCCACATACTCTGATGATCCCCAATCGTGGCGTGTCGGCCTGCTTCGGCAGGCACTGGCGCCGGGATCATTCATGGCAA
P6	GCTCGCTTTCTTGCTGTCCAATTTCTATTAAAGGTTCCTTTGTTCCCTAAGTCCAACTACTAAACTGGGGGATATTATGAAGGGCCTTGAGCATCTGGATTCTGCCTAATAAAAAACA	GCTCGCTTTCTTGCTGTCCAATTTCTATTAAAGGTTCCTTTGTTCCCTAAGTCCAACTACTAAACTGGGGGATATTATGAAGGGCCTTGAGCATCTGGATTCTGCCTAATAAAAAACATTTATTTTCATTGCAATTGCCATGTGTATGTGGGTTCGCCCACATACTCTGATGATCCCCAATCGTGGCGTGTCGGCCTGCTTCGGCAGGCACTGGCGCCGGGATCATTCATGGCAA
P7	ATTAAAGGTTTATACCTTCCCAGGTAGCAAAACCAACCAACTCTCGATCTCTTGTAGATCTGTTCTCTAAACGAACTTTAAAATCTGTGTGGCTGTCACTTGGCTGCATGCCTAGTGCACTCACGCAGTATAATAATAATTAATTACTGTCGTTGACAGGAAACGAGTAACTCGTCCGTCTTCTGCAGACTGCTTACGGTTTCGTCCGTGTTGCAGTCGATCATCAGCATACCTAGGTTTTGTCCGGGTGTGACCGAAAGGTAAG	GCTCGCTTTCTTGCTGTCCAATTTCTATTAAAGGTTCCTTTGTTCCCTAAGTCCAACTACTAAACTGGGGGATATTATGAAGGGCCTTGAGCATCTGGATTCTGCCTAATAAAAAACATTTATTTTCATTGCAATTGCCATGTGTATGTGGGTTCGCCCACATACTCTGATGATCCCCAATCGTGGCGTGTCGGCCTGCTTCGGCAGGCACTGGCGCCGGGATCATTCATGGCAA
P8	ACCGCCGAGACCGCGTCCGCCCCGCGAGCACAGAGCCTCGCCTTTGCCGATCCGCCGCCCGTCCACACCCGCCGCCAGCTCACC	AACTTGTTTATTGCAGCTTATAATGGTTACAAATAAAGCAATAGCATCACAAATTTCACAAATAAAGCATTTTTTTCACTGCATTCTAGTTGTGGTTTGTCCAAACTCATCAATGTATCTTA

P9	ACCGCCGAGACCGCGTCCGCCCCGCGAGCACAGAGCCTCGCCTTTGCCGATCCGCCGCCCGTCCACACCCGCCGCCAGCTCACC	ATACAGCAGCAATTGGCAAGCTGCTTACATAGAACTCGCGGCGATTGGCATGCCGCCTTAAAATTTTTATTTTATTTTTCTTTTCTTTTCCGAATCGGATTTTGTTTTTAATATTTC
P10	ACCGCCGAGACCGCGTCCGCCCCGCGAGCACAGAGCCTCGCCTTTGCCGATCCGCCGCCCGTCCACACCCGCCGCCAGCTCACC	CATGGTTGTACTGCACTATCATCCTAAGACGGTCCTTCTTCGGATCGCAATCTCACCCTGGTGCCGCGCTTCCTTCGGGAACTGCACCCGCGGACCAGGGCCGTCTTTGAACTTTTCTAACTGTTCTTAC
P11	ACCGCCGAGACCGCGTCCGCCCCGCGAGCACAGAGCCTCGCCTTTGCCGATCCGCCGCCCGTCCACACCCGCCGCCAGCTCACC	GATTAGAATTTCAGAAATATGAGGAAAAACACCCTTGTTTCTACTGATTAGAATTTCAGAAATATGAGGAAAAACACCCTTGTTTCTACT
P12	ACCGCCGAGACCGCGTCCGCCCCGCGAGCACAGAGCCTCGCCTTTGCCGATCCGCCGCCCGTCCACACCCGCCGCCAGCTCACC	AGAAAGTTTTCAATTTGGCTTTAATTCGGTTTAATTGGAAGACCCTGTCACGTATCTGGACGTGACAAAAGCGTGACAGGGGTAAATTTAGCACAACCAGAGTGCT
P13	ACCGCCGAGACCGCGTCCGCCCCGCGAGCACAGAGCCTCGCCTTTGCCGATCCGCCGCCCGTCCACACCCGCCGCCAGCTCACC	TAACCCTACCTCAGTCGAATTGGATTGGGTTATACTGTTGTAGGGGTAAATTTTTCTTTAATTCGGAGA
P14	ACCGCCGAGACCGCGTCCGCCCCGCGAGCACAGAGCCTCGCCTTTGCCGATCCGCCGCCCGTCCACACCCGCCGCCAGCTCACCGGCTAGCGCCGCCACC	GCTCGCTTTCTTGCTGTCCAATTTCTATTAAAGGTTCCTTTGTTCCCTAAGTCCAACTACTAAACTGGGGGATATTATGAAGGGCCTTGAGCATCTGGATTCTGCCTAATAAAAAACATTTATTTTCATTGCAATTGCCATGTGTATGTGGGTTCGCCCACATACTCTGATGATCCCCAATCGTGGCGTGTCGGCCTGCTTCGGCAGGCACTGGCGCCGGGATCATTCATGGCAA
P15	ACCGCCGAGACCGCGTCCGCCCCGCGAGCACAGAGCCTCGCCTTTGCCGATCCGCCGCCCGTCCACACCCGCCGCCAGCTCACC	AAAGCAAAACTAACATGAAACAAGGCTAGAAGTCAGGTCGGATTAAGCCATAGTACGGAAAAAACTATGCTACCTGTGAGCCCCGTCCAAGGACGTTAAAAGAAGTCAGGCCATCATAAATGCCATAGCTTGAGTAAACTATGCAGCCTGTAGCTCCACCTGAGAAGGTGTAAAAAATCCGGGAGGCCACAAACCATGGAAGCTGTACGCATGGCGTAGTGGACTAGCGGTTAGAGGAGACCCCTCCCTTACAAATCGCAGCAACAATGGGGGCCCAAGGCGAGATGAAGCTGTAGTCTCGCTGGAAGGACTAGAGGTTAGAGGAGACCCCCCCGAAACAAAAAACAGCATATTGACGCTGGGAAAGACCAGAGATCCTGCTGTCTCCTCAGCATCATTCCAGGCACAGAACGCCAGAAAATGGAATGGTGCTGTTGAATCAACAGGTTCT
P16	ACCGCCGAGACCGCGTCCGCCCCGCGAGCACAGAGCCTCGCCTTTGCCGATCCGCCGCCCGTCCACACCCGCCGCCAGCTCACC	CCCCTCCCTTACAAATCGCAGCAACAATGGGGGCCCAAGGCGAGATGAAGCTGTAGTCTCGCTGGAAGGACTAGAGGTTAGAGGAGACCCCCCCGAAACAAAAAACAGCATATTGACGCTGGGAAAGACCAGAGATCCTGCTGTCTCCTCAGCATCATTCCAGGCACAGAACGCCAGAAAATGGAATGGTGCTGTTGAATCAACAGGTTCT
P17	ACCGCCGAGACCGCGTCCGCCCCGCGAGCACAGAGCCTCGCCTTTGCCGATCCGCCGCCCGTCCACACCCGCCGCCAGCTCACC	GTTCTCTTCCAGTTTGCAGGTGTTCCTGTGACCCTGACACCCTCCTTCTGCACATGGGGACTGGGCTTGGCCTTGAGAGAAAGCCTTCTGTTTAATAAAGTACATTTTCTTCAGTAATCAAAAA
P18	ACCGCCGAGACCGCGTCCGCCCCGCGAGCACAGAGCCTCGCCTTTGCCGATCCGCCGCCCGTCCACACCCGCCGCCAGCTCACC	GATCCTGGACTGTTTCCTGATAACCATAAGAAGACCCTATTTCCCTAGATTCTATTTTCTGAACTTGGGAACACAATGCCTACTTCAAGGGTATGGCTTCTGCCTAATAAAGAATGTTCAGCTCAA
P19	ACCGCCGAGACCGCGTCCGCCCCGCGAGCACAGAGCCTCGCCTTTGCCGATCCGCCGCCCGTCCACACCCGCCGCCAGCTCACC	ACTCATGCAGACCACACAAGGCAGATGGGCTATGTAAACGTTTTCGCTTTTCCGTTTACGATACATAGTCTACTCTTGTGCAGAATGAAATCTCGTAGCTATACAGCACAAGTAGGTATAGTTAACTTTAATCTCACATAGCAATCTTTAATCAGTGTGTAACATTAGGGAGGACTTGAAAGAGCCACCACATTTTCACCGAGGCCACGCGGAGTACGATCGAGGGTACAGTGAATAATGCTAGGGAGAGCTGCCTATATGGAAGAGCCCTAATGTGTAAAATTAATTTTAGTAGTGCTATCCCCATGTGATTTTAATAGCTTCTTAGGAGAATGACAA
PolyA-1	AAAAAAAAAAAAAAAAAAAAAAAAAAAAAAAAAAAAAAAAAAAAAAAAAAAAAAAAAAAA	

PolyA-2	AAAAAAAAAAAAAAAAAAAAAAAAAAAAAAAAAAAAAAAAAAAAAAAAAAAAAAAAAAAAAAAAA AAAAAAAAAAAAAAA
PolyA-3	AAAAAAAAAAAAAAAAAAAAAAAAAAAAAAAAAAAAAAAAAAAAAAAAAAAAAAAAAAAAAAAAA AAAAAAAAAAAAAAAAAAAAAAAAAAAAAAAAAAA
PolyA-4	AAAAAAAAAAAAAAAAAAAAAAAAAAAAAAAAAAAAAAAAAAAAAAAAAAAAAAAAAAAAAAAAA AAAAAAAAAAAAAAAAAAAAAAAAAAAAAAAAAAAAAAAAAAAAAAAAAAAAAAA
PolyA-5	AAAAAAAAAAAAAAAAAAAAAAAAAAAAAAAAAAAAAAAACTGGATTCTGAAAAAAAAAAAAAAAA AAAAAAAAAAAAAAAAAAAAAAAAAAAAAAAAAAAAAAAAAAAA

Table S2: sequences for the RV-G RNA

NO.	SEQ.
RV-G-1	GGGGCGCUGCCUACGGAGGUGGCAGCCAUCUCCUUCUCGGCAUCGGAUCCGCCACCAUGA UCCCUCAGGCCCUGCUGUUCGUGCCCCUGCUGGUGUUUCCUCUGUGCUUCGGCAAGUUC CCAAUCUACACCAUCCCAGAUAAGCUGGGACCUUGGAGCCCUAUCGACAUUCACCACCUGA GUUGUCCUAACAACCUGGUGGUCGAGGACGAGGGCUGUACCAAUCUGAGCGGCUUCUCC UACAUGGAACUGAAGGUGGGCUACAUCAGCGCCAUCAAGGUGAACGGCUUCACUUGCACC GGCGUGGUGACCGAGGCCGAGACAUACACAAAUUUCGUUGGCUACGUGACCACCACCUUC AAGAGAAAGCACUUCCGCCCCACCCCUGACGCCUGUCGGAGCGCUUACAACUGGAAGAUG GCCGGCGAUCCUAGAUACGAGGAAAGUCUGCACAACCCCUAUCCUGAUUACCACUGGCUG AGAACCGUGAAGACCACAAAGGAAAGCGUGGUGAUCAUUAGCCCUAGCGUGGCCGAUCUG GACCCUUACGACAAGUCUCUCCAUAGCAGAGUGUUCCCUAGAGGCAAGUGCAGCGGCAUA ACAGUGAGCUCCGCCUACUGCAGCACCAAUCACGACUACACCAUCUGGAUGCCUGAGAACC CUAGACUGGGUACAUCUUGUGAUAUCUUCACAAACAGCAGAGGCAAACGGGCUUCUAAA GGCAGCAAGACCUGUGGCUUUGUGGACGAGCGGGGACUGUACAAAUCUCUGAAGGGCGC CUGCAAGCUGAAACUGUGCGGCGUGCUGGGCCUCCGGCUGAUGGACGGCACCUGGGUCG CCAUCCAGACCAGCAACGAGACAAAGUGGUGCCCCCCGAUCAGCUGGUGAAUCUGCACGA UUUCCACAGCGACGAAAUCGAGCAUCUGGUGGUGGAAGAACUGGUUAAAAAGCGGGAAG AGUGCCUGGAUGCUCUGGAAAGCAUCAUGACAACCAAAUCCGUGAGCUUCCGGAGGCUGA GCCACCUGAGAAAGCUGGUUCCCGGCUUCGGCAAGGCCUAUACCAUCUUUAACAAGACAC UGAUGGAAGCCGACGCCCACUACAAGAGCGUCCGGACCUGGAACGAGAUCAUCCCUAGCA AGGGCUGCCUGAGAGUGGGCGGAAGAUGCCACCCCCACGUGAACGGCGUGUUUUUCAAC GGCAUCAUCCUGGGCCCUGACGGCCACGUGCUGAUCCCUGAGAUGCAGUCCAGCCUGCUG CAGCAGCACAUGGAACUGCUGGAGAGCUCUGUGAUCCCCCUGAUGCACCCUCUGGCCGAC CCCAGCACAGUGUUUAAGGACGGCGACGAGGUGGAGGACUUCGUGGAAGUGCACCUGCC UGACGUGCAUAAGCAGGUGAGCGGCGUGGACCUGGGACUGCCAAACUGGGGAAAAGACG UGCUGAUGGGCGCUGGCGUGCUGACCGCCCUGAUGCUGAUGAUCUUCCUGAUGACAUGC UGCAGAAGAACCAACCGGGCCGAGAGCAUCCAACACAGCCUGGGCGAGACCGGCAGAAAGG UGUCGGUCACCUCUCAGUCUGGAAGAGUGAUCAGCUCUUGGGAGAGCUACAAGAGCGGA GGAGAAACCAAACUGUGAUAACUCGAGCAUCACAUUUAAAAGCAUCUCAGCCUACCAUGA GAAUAAGAGAAAGAAAAUGAAGAUCAAUAGCUUAUUCAUCUCUUUUUCUUUUUCGUUG GUGUAAAGCCAACACCCUGUCUAAAAAACAUAAAUUUCUUUAAUCAUUUUGCCUCUUUU CUCUGUGCUUCAAUUAAUAAAAAAUGGAAAGAACCUAAAAAAAAAAAAAAAAAAAAAAAAA AAAAAAAAAAAAAAAAAAAAAAAAAAAAAAAAAAAAAAAAUGCAUCCCCCCCCCCCCCCC CCCCCCCCCCCCCCCCCCCCAAAGGCUCUUUUCAGAGCCACCA
RV-G-2	AAAAUCCGUUGACCUUAAACGGUCGUGUGGGUUCAAGUCCCUCCACCCCCACGCCGGAAA CGCAAUAGCCGAAAAACAAAAAACAAAAAAAACAAAAAAAAAACCAAAAAAACAAAACACAU UAAAACAGCCUGUGGGUUGAUCCCACCCACAGGCCCAUUGGGCGCUAGCACUCUGGUAUC ACGGUACCUUUGUGCGCCUGUUUUAUACCCCCUCCCCCAACUGUAACUUAGAAGUAACAC ACACCGAUCAACAGUCAGCGUGGCACACCAGCCACGUUUUGAUCAAGCACUUCUGUUACC CCGGACUGAGUAUCAAUAGACUGCUCACGCGGUUGAAGGAGAAAGCGUUCGUUAUCCGG CCAACUACUUCGAAAAACCUAGUAACACCGUGGAAGUUGCAGAGUGUUUCGCUCAGCACU

	ACCCCAGUGUAGAUCAGGUCGAUGAGUCACCGCAUUCCCCACGGGCGACCGUGGCGGUGG<br>CUGCGUUGGCGGCCUGCCCAUGGGGAAACCCAUGGGACGCUCUAAUACAGACAUGGUGCG<br>AAGAGUCUAUUGAGCUAGUUGGUAGUCCUCCGGCCCCUGAAUGCGGCUAAUCCUAACUG<br>CGGAGCACACACCCUCAAGCCAGAGGGCAGUGUGUCGUAACGGGCAACUCUGCAGCGGAA<br>CCGACUACUUUGGGUGUCCGUGUUUCAUUUUAUUCCUAUACUGGCUGCUUAUGGUGAC<br>AAUUGAGAGAUCGUUACCAUAUAGCUAUUGGAUUGGCCAUCCGGUGACUAAUAGAGCUA<br>UUAUAUAUCCCUUUGUUGGGUUUAUACCACUUAGCUUGAAAGAGGUUAAAACAUUACAA<br>UUCAUUGUUAAGUUGAAUACAGCAAAAUGAUCCCUCAGGCCCUGCUGUUCGUGCCCCUGC<br>UGGUGUUUCCUCUGUGCUUCGGCAAGUUCCCAAUCUACACCAUCCCAGAUAAGCUGGGAC<br>CUUGGAGCCCUAUCGACAUUCACCACCUGAGUUGUCCUAACAACCUGGUGGUCGAGGACG<br>AGGGCUGUACCAAUCUGAGCGGCUUCUCCUACAUGGAACUGAAGGUGGGCUACAUCAGC<br>GCCAUCAAGGUGAACGGCUUCACUUGCACCGGCGUGGUGACCGAGGCCGAGACAUACACA<br>AAUUUCGUUGGCUACGUGACCACCACCUUCAAGAGAAAGCACUUCCGCCCCACCCCUGACG<br>CCUGUCGGAGCGCUUACAACUGGAAGAUGGCCGGCGAUCCUAGAUACGAGGAAAGUCUGC<br>ACAACCCCUAUCCUGAUUACCACUGGCUGAGAACCGUGAAGACCACAAAGGAAAGCGUGG<br>UGAUCAUUAGCCCUAGCGUGGCCGAUCUGGACCCUUACGACAAGUCUCUCCAUAGCAGAG<br>UGUUCCCUAGAGGCAAGUGCAGCGGCAUAACAGUGAGCUCCGCCUACUGCAGCACCAAUC<br>ACGACUACACCAUCUGGAUGCCUGAGAACCCUAGACUGGGUACAUCUUGUGAUAUCUUCA<br>CAAACAGCAGAGGCAAACGGGCUUCUAAAGGCAGCAAGACCUGUGGCUUUGUGGACGAGC<br>GGGGACUGUACAAAUCUCUGAAGGGCGCCUGCAAGCUGAAACUGUGCGGCGUGCUGGGC<br>CUCCGGCUGAUGGACGGCACCUGGGUCGCCAUCCAGACCAGCAACGAGACAAAGUGGUGC<br>CCCCCCGAUCAGCUGGUGAAUCUGCACGAUUUCCACAGCGACGAAAUCGAGCAUCUGGUG<br>GUGGAAGAACUGGUUAAAAAGCGGGAAGAGUGCCUGGAUGCUCUGGAAAGCAUCAUGAC<br>AACCAAAUCCGUGAGCUUCCGGAGGCUGAGCCACCUGAGAAAGCUGGUUCCCGGCUUCGG<br>CAAGGCCUAUACCAUCUUUAACAAGACACUGAUGGAAGCCGACGCCCACUACAAGAGCGUC<br>CGGACCUGGAACGAGAUCAUCCCUAGCAAGGGCUGCCUGAGAGUGGGCGGAAGAUGCCAC<br>CCCCACGUGAACGGCGUGUUUUUCAACGGCAUCAUCCUGGGCCCUGACGGCCACGUGCUG<br>AUCCCUGAGAUGCAGUCCAGCCUGCUGCAGCAGCACAUGGAACUGCUGGAGAGCUCUGUG<br>AUCCCCCUGAUGCACCCUCUGGCCGACCCCAGCACAGUGUUUAAGGACGGCGACGAGGUG<br>GAGGACUUCGUGGAAGUGCACCUGCCUGACGUGCAUAAGCAGGUGAGCGGCGUGGACCU<br>GGGACUGCCAAACUGGGGAAAAGACGUGCUGAUGGGCGCUGGCGUGCUGACCGCCCUGA<br>UGCUGAUGAUCUUCCUGAUGACAUGCUGCAGAAGAACCAACCGGGCCGAGAGCAUCCAAC<br>ACAGCCUGGGCGAGACCGGCAGAAAGGUGUCGGUCACCUCUCAGUCUGGAAGAGUGAUCA<br>GCUCUUGGGAGAGCUACAAGAGCGGAGGAGAAACCAAACUGUGAUAAAAAAAACAAAAAA<br>CAAAACGGCUAUUAUGCGUUACCGGCGAGACGCUACGGACUU
RV-G-3	GAGGACCGCCGAGACCGCGUCCGCCCCGCGAGCACAGAGCCUCGCCUUUGCCGAUCCGCCG<br>CCCGUCCACACCCGCCGCCAGCUCACCGGAUCCGCCACCAUGAUCCCUCAGGCCCUGCUGU<br>UCGUGCCCCUGCUGGUGUUUCCUCUGUGCUUCGGCAAGUUCCCAAUCUACACCAUCCCAG<br>AUAAGCUGGGACCUUGGAGCCCUAUCGACAUUCACCACCUGAGUUGUCCUAACAACCUGG<br>UGGUCGAGGACGAGGGCUGUACCAAUCUGAGCGGCUUCUCCUACAUGGAACUGAAGGUG<br>GGCUACAUCAGCGCCAUCAAGGUGAACGGCUUCACUUGCACCGGCGUGGUGACCGAGGCC<br>GAGACAUACACAAAUUUCGUUGGCUACGUGACCACCACCUUCAAGAGAAAGCACUUCCGC<br>CCCACCCCUGACGCCUGUCGGAGCGCUUACAACUGGAAGAUGGCCGGCGAUCCUAGAUAC<br>GAGGAAAGUCUGCACAACCCCUAUCCUGAUUACCACUGGCUGAGAACCGUGAAGACCACA<br>AAGGAAAGCGUGGUGAUCAUUAGCCCUAGCGUGGCCGAUCUGGACCCUUACGACAAGUC<br>UCUCCAUAGCAGAGUGUUCCCUAGAGGCAAGUGCAGCGGCAUAACAGUGAGCUCCGCCUA<br>CUGCAGCACCAAUCACGACUACACCAUCUGGAUGCCUGAGAACCCUAGACUGGGUACAUC<br>UUGUGAUAUCUUCACAAACAGCAGAGGCAAACGGGCUUCUAAAGGCAGCAAGACCUGUGG<br>CUUUGUGGACGAGCGGGGACUGUACAAAUCUCUGAAGGGCGCCUGCAAGCUGAAACUGU<br>GCGGCGUGCUGGGCCUCCGGCUGAUGGACGGCACCUGGGUCGCCAUCCAGACCAGCAACG<br>AGACAAAGUGGUGCCCCCCCGAUCAGCUGGUGAAUCUGCACGAUUUCCACAGCGACGAAA<br>UCGAGCAUCUGGUGGUGGAAGAACUGGUUAAAAAGCGGGAAGAGUGCCUGGAUGCUCUG<br>GAAAGCAUCAUGACAACCAAAUCCGUGAGCUUCCGGAGGCUGAGCCACCUGAGAAAGCUG

	GUUCCCGGCUUCGGCAAGGCCUAUACCAUCUUUAACAAGACACUGAUGGAAGCCGACGCC<br>CACUACAAGAGCGUCCGGACCUGGAACGAGAUCAUCCCUAGCAAGGGCUGCCUGAGAGUG<br>GGCGGAAGAUGCCACCCCCACGUGAACGGCGUGUUUUUCAACGGCAUCAUCCUGGGCCCU<br>GACGGCCACGUGCUGAUCCCUGAGAUGCAGUCCAGCCUGCUGCAGCAGCACAUGGAACUG<br>CUGGAGAGCUCUGUGAUCCCCCUGAUGCACCCUCUGGCCGACCCCAGCACAGUGUUUAAG<br>GACGGCGACGAGGUGGAGGACUUCGUGGAAGUGCACCUGCCUGACGUGCAUAAGCAGGU<br>GAGCGGCGUGGACCUGGGACUGCCAAACUGGGGAAAAGACGUGCUGAUGGGCGCUGGCG<br>UGCUGACCGCCCUGAUGCUGAUGAUCUUCCUGAUGACAUGCUGCAGAAGAACCAACCGGG<br>CCGAGAGCAUCCAACACAGCCUGGGCGAGACCGGCAGAAAGGUGUCGGUCACCUCUCAGU<br>CUGGAAGAGUGAUCAGCUCUUGGGAGAGCUACAAGAGCGGAGGAGAAACCAAACUGUGA<br>UAACUCGAGGCUCGCUUUCUUGCUGUCCAAUUUCUAUUAAAGGUUCCUUUGUUCCCUAA<br>GUCCAACUACUAAACUGGGGGAUAUUAUGAAGGGCCUUGAGCAUCUGGAUUCUGCCUAA<br>UAAAAAACAUUUAUUUUCAUUGCAAUUGCCAUGUGUAUGUGGGUUCGCCCACAUACUCU<br>GAUGAUCCCCAAUCGUGGCGUGUCGGCCUGCUUCGGCAGGCACUGGCGCCGGGAUCAUUC<br>AUGGCAAAAAAAAAAAAAAAAAAAAAAAAAAAAAAAAAAAAAAAAAACUGGAUUCUGAAA<br>AAAAAAAAAAAAAAAAAAAAAAAAAAAAAAAAAAAAAAAAAAAAAAAAAAAAAA

Figure S1: Original images for WB

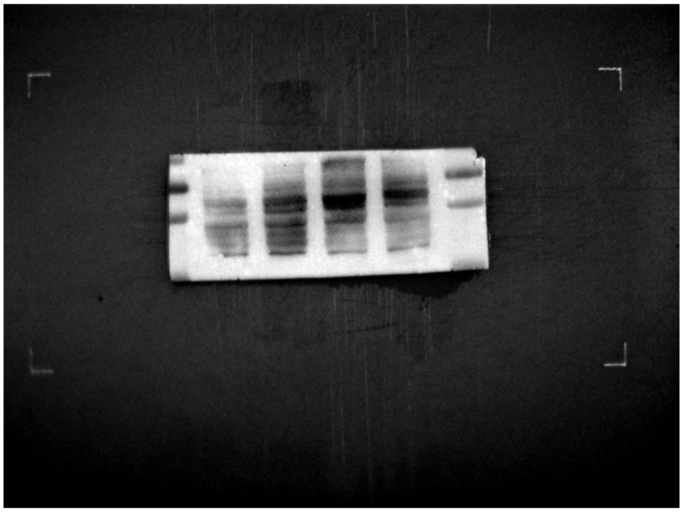

RV-G

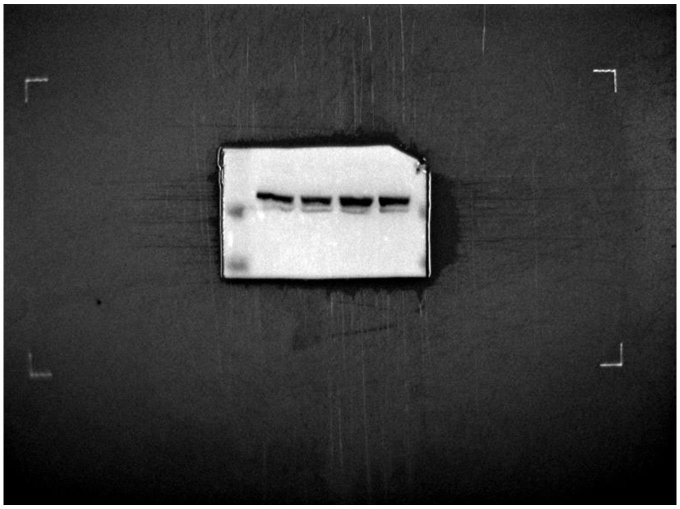

GAPDH
